# Photodynamic Effect of Methylene Blue and Low Level Laser Radiation in Head and Neck Squamous Cell Carcinoma Cell Lines

**DOI:** 10.3390/ijms19041107

**Published:** 2018-04-07

**Authors:** Barbara Kofler, Angela Romani, Christian Pritz, Teresa Bernadette Steinbichler, Volker Hans Schartinger, Herbert Riechelmann, Jozsef Dudas

**Affiliations:** Department of Otorhinolaryngology, Medical University of Innsbruck, 6020 Innsbruck, Austria; angela.romani@tirol-kliniken.at (A.R.); christianpritz@gmail.com (C.P.); teresa.steinbichler@tirol-kliniken.at (T.B.S.); volker.schartinger@i-med.ac.at (V.H.S.); herbert.riechelmann@i-med.ac.at (H.R.); jozsef.dudas@i-med.ac.at (J.D.)

**Keywords:** photodynamic therapy, methylene blue, head and neck carcinoma, TissueFaxs

## Abstract

Photodynamic therapy (PDT) is suggested to have an impact on the treatment of early stage head and neck cancers (HNSCC). We investigated the effect of PDT with methylene blue (MB) and a diode laser (660 nm) as the laser source on HNSCC cell lines as an in vitro model of surface oral squamous cell carcinoma. Cell-cultures were exposed to 160 µM MB for 4 min and to laser light for 8 min. Viability was proven via cell viability assay and clonogenic survival via clone counting assay. The combination of MB and diode laser evidenced high efficient loss of cell viability by 5% of the control, while treatment with the same concentration of MB for 4 min alone showed a viability of 46% of the control. In both SCC-25 and Detroit 562 HNSCC cells, MB combined with the laser allowed a significant abrogation of clonogenic growth (*p* < 0.01), especially in the case of Detroit 562 cells less than 1% of the suspension plated cells were able to grow tumor cell nests. Multiresistant (Detroit 562) HNSCC cells expressing cancer stem cell markers are sensitive to MB/red laser combined PDT.

## 1. Introduction

Head and neck malignancies compose about 90% of squamous cell carcinoma (SCC). Depending on several factors including stage of cancer and condition of the patient, different treatment options are available. Main treatment principles include surgery, radiotherapy, chemotherapy, immune therapy, or combinations thereof [1,2,3]. Occasionally, other treatment modalities may be beneficial in selected patients.

Photodynamic therapy (PDT) is a treatment modality for precancerous skin lesions with good cosmetic results and few adverse effects and allows application to multiple lesions [4]. Several studies reported successful treatment of early stage head and neck squamous cell carcinoma (HNSCC) and its precursor lesions with photodynamic therapy (PDT) [5,6].

Recently, an overall response rate of 91% with a complete response rate of 71% in early stage oral cavity and oropharyngeal cancer was observed in 170 HNSCC-patients. In these studies, the photosensitizer meta-tetrahydroxyphenylchlorin (mTHPC) was combined with a diode laser at 652 nm for PDT [7]. Rigual and coworker [8] assessed the response of dysplasia, intraepithelial neoplasia, and T1 carcinomas of the oral cavity to PDT with porfimer sodium and light at 630 nm. Responses were evaluated at 1 week, 1 month, and 3 months following intervention. Initially, 25 patients had a complete response, one a partial response, and one patient did not respond. Three patients with oral dysplasia with an initial complete response had a recurrence in the treatment field. The authors concluded that PDT with porfimer sodium is an effective treatment for these indications [8]. PDT as possible treatment modality in high risk dysplasia, intraepithelial neoplasia, and T1 SCC of the larynx was investigated in a single institution, Phase-Ib study on pyropheophorbide (HPPH)-PDT. In this study, 29 subjects and 30 lesions were treated. Patients with T1 SCC had a complete response rate of 82%. The most common adverse event was transient hoarseness. Two patients required tracheostomy because of laryngeal edema [9].

### 1.1. Mechanisms of PDT and Common Photosensitizer

Photodynamic therapy (PDT) requires three components, the photosensitizer, oxygen, and light source [4]. The basic principle of PDT is energy transfer from the photosensitizer to oxygen in the tissue under light excitation. Under light excitation, reactive oxygen species, especially singlet oxygen, are generated which cause cellular toxicity. Selective uptake of the photosensitizer in premalignant or malignant cells is the clue to specificity of PDT [10]. Repeatability is an important feature of PDT, furthermore PDT has no accumulative destructive effect and does not negatively influence further treatment like radiation or chemotherapy [7]. A variety of photosensitizers are available [11,12].

5-aminolevulinic acid hydrochloride (5-ALA) is a porphyrin, which gets metabolized in the target tissue to the proper photosensitizer. The porphyrin 5-ALA accumulates selectively in tumor tissues and is rapidly eliminated from the human body. It is routinely used for PDT treatment of actinic keratosis [13]. Another common photosensitizer is mTHPC, a lipophilic chlorin of the second generation, which has a high tumor selectivity and a high yield of reactive oxygen species [11,14,15].

### 1.2. Methylene Blue as A Photosensitizer

Methylene blue (MB), also known as methylthioninium chloride, is a hydrophilic phenothiazine derivative. It is a photosensitizer with light absorption at 660 nm [16]. This maximum lies well within the emission range of common diode lasers used for low level laser therapy. These lasers are frequently at hand at institutions treating head & neck cancer patients or are available at low cost [17]. Environmental light does barely activate MB. Therefore, adverse effects due to environmental light exposure are not to be expected. Moreover, MB is an inexpensive photosensitizer.

MB is used for antimicrobial photodynamic therapy (APDT) and is used as a potent PDT drug for local treatment of periodontal diseases [15], because of its efficiency against a broad spectrum of microbes including bacteria, fungi, and viruses [18,19]. The efficiency of MB-APDT has also been demonstrated on an antibiotic resistant polymicrobial biofilms of *Pseudomonas aeruginosa* and *Methicillin-resistant Staphylococcus aureus* (MRSA) in a maxillary sinus model. An in vitro maxillary sinus biofilm study demonstrated that APDT reduced the polymicrobial biofilm in chronic rhinosinusitis by >99.99% after a single treatment [20]. Different MB concentration and exposure times were reported. Betsy and coworker assessed 90 patients with untreated chronic periodontitis for scaling and root planning and APDT or scaling and root planning alone. The photosensitizer used consisted of MB suspended in double distilled water at a concentration of 10 mg/mL. As light source a diode laser operating at 655 nm was used [21]. MB concentrations used in clinical studies ranged from 100 µg/mL [22] to 10 µg/mL [23].

A Brazilian study group proved PDT in pediatric dentistry. APDT was performed using methylene blue (50 µg/mL) as photosensitizer for 5 min as pre irradiation time and after the red laser was delivered [24]. Another Brazilian study group used PDT with methylene blue for onychomycosis. MB 2% aqueous solution was applied to the lesion until saturation occurred, followed by a rest period of 3 min. The MB solution was not washed off. After the rest period, the lesion was immediately illuminated with noncoherent red light (630 nm) [25]. Early reports suggest that tumor selectivity of MB is low. Direct application of MB on the tumor site may result in accumulation within tumor cells. In analogy to toluidine blue, this effect is probably due to impaired epithelial barrier at the tumor site. In order to improve tumor cell selectivity, MB has been targeted specifically to tumor cells. Therefore, MB was embedded into a nanoparticle carrying tumor antibodies or tumor-specific peptides [26,27,28]. Recently Fan et al. [29] reported about the development of MB bound nanoplatform, which is capable of delivering targeted diagnostic and photodynamic treatment of cancer. Once the nanoparticle binds with the target cell surface, it can detect human prostate cancer cell selectively using fluorescence imaging and PDT treatment using 785 nm, near infrared light indicates that the multimodal treatment increases the possibility of destroying prostate cancer cells in vitro [29].

### 1.3. In Vitro Data

There exist various in vitro studies of PDT on different cell lines using various photosensitizers. El-Khatib and coworker [30] examined the effect of PDT with 5-ALA in primary meningioma cell lines. For PDT, about 5000 cells per well were plated in 20 wells of a blank 96-well plate. In each block of four wells, 150 μL of 0, 25, 50, and 100 μg/mL 5-ALA solutions was inoculated and one block was used as the negative control without 5-ALA and without light application. PDT was performed for 3 h using a laser (635 nm, 18.75 J/cm^2^). A cell viability assay was performed 90 min after PDT. The authors observed a significant and dose-dependent decrease of viability. Either 5-ALA or PDT alone did not affect viability [30]. Mirzaei and coworker [31] evaluated the photodynamic effect with radachlorin as photosensitizer on human liver cancer cells (HepG2) and normal liver cells (HFLF-PI4) measuring the viability using the 3-(4,5-dimethylthiazol-2-yl)-2,5-diphenyltetrazolium bromide) tetrazolium (MTT) assay. The photosensitizer radachlorin without light irradiation had no toxic effect on the cell lines. Cell survival of HepG2 and HFLF-PI4 cells were decreased following PDT in a concentration-dependent manner. The study group could also observe that the HepG2 cells were more sensitive to radachlorin PDT than HFLF-PI4 cells. The 50% lethal dose of radachlorin HepG2 cells were 30 μg/mL and 20 μg/mL, 24 h after exposure to doses of 5 J/cm^2^ and 15, or 25 J/cm^2^. To kill HepG2 cells with minimal effects on normal HFLF-PI4 cells the optimal radachlorin and light dose were 100 μg/mL and 15 J/cm^2^ [31]. Another study group investigated the potential use of 5-ALA PDT induced protoporphyrin IX (PPIX) on a nasopharyngeal carcinoma cell line. The cells were irradiated at 4 h after incubation with 5-ALA (10–200 μg/mL) by a diode laser (630 nm) at various energy levels (1–50 J/cm^2^). After incubation with 5-ALA, a time- and dose-dependent increase of cellular PPIX-fluorescence was seen up to a threshold concentration of 1000 μg/mL 5-ALA. The combination of 5-ALA and laser irradiation leaded to a significant, concentration-, energy-, and time-dependent increase of cell death. Cellular survival at 100 μg/mL ALA and 10 J/cm^2^ laser irradiation was <5% after 48 h [32]. Guan and coauthors [33] evaluated PDT with MB on an osteosarcoma-derived cell line (UMR 106). The photocytotoxicity on the cell line was investigated 24 h after MB PDT using sulforhodamine B assay (SRB) and light microscopy. MB under red light irradiation caused a drug-concentration (0–100 μM) and light-dose (0–32 J/cm^2^) dependent cytotoxicity. Furthermore, the SRB assay and light microscopy showed a significant decrease of cell numbers after LED light-activated MB treatment (100 μM, 32 J/cm^2^) [33].

It is particularly interesting to investigate the effect of PDT in therapy resistant tumor cells in HNSCC. For this purpose, two cell lines have been included in this study. SCC-25 cells were originally isolated from the primary tumor of a patient with tongue carcinoma [34,35]. SCC-25 cells formed tumors in severe combined immunodeficiency (SCID) mice but not in athymic nude mice suggesting less aggressive behavior. Moreover, SCC-25 induced tumors did not develop regional or distant metastasis in mouse models [36]. In vitro, SCC-25 cells were found to be radioresistant [37]. SCC-25 cells contain a deletion and a frame shift in the *TP53* gene protein coding region and do not synthesize any p53 protein (own Sanger sequencing results). This cell line represents a typical issue also common in HNSCC patients, the p53 protein loss. Detroit 562 cells grow tumors and develop regional and lung metastases when injected in nude mice [38]. Detroit 562 was isolated from the malignant pleural effusion of a patient with pharyngeal carcinoma [39,40]. A frequent gain of function mutation R175H of *TP53* gene is contained in Detroit 562 cells [41], which has been confirmed by us utilizing Sanger sequencing. Both SCC-25 and Detroit 562 cells were HPV-negative [41].

In the present study, we examined the photodynamic effect on these two HNSCC cell lines using methylene blue as photosensitizer and a diode laser at 660 nm at a fixed area dose of 95 J/cm^2^. We aimed to identify MB concentrations, MB exposure times and laser exposure times in order to achieve maximum tumor toxicity due to phototoxic effect at minimum tumor toxicity due to MB alone.

## 2. Results

### 2.1. Stem Cell Characteristics in the SCC-25 and Detroit 562 Cell Lines

**Figure 1:**
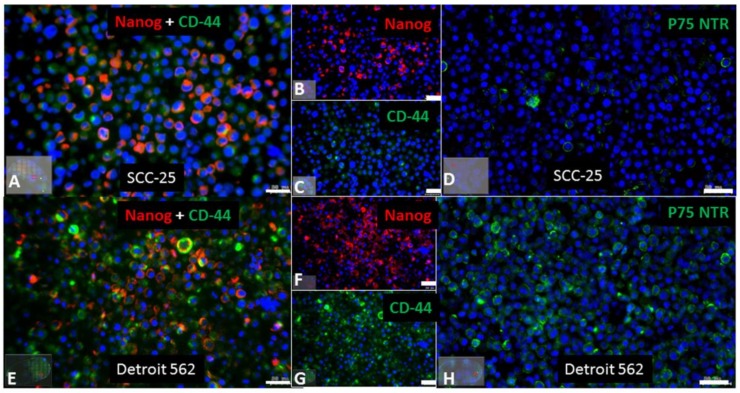
Detection of cancer stem cell markers in SCC-25 and Detroit 562 cells. SCC-25 (**A**–**D**) and Detroit 562 (**E**–**H**) cells have been cultured to confluency, collected in pellets, embedded in agarose and in paraffin, sectioned and were subjected to combined indirect immunohistochemical reactions for Nanog (detected in red) and CD-44 (detected in green) (**A**–**G**) and to single immunohistochemical reaction for p75NTR (detected in green; **D**,**H**). Detroit 562 cells showed more frequent reactions for all cancer stem cell markers. Scale bars: 50 µm.

P75 neurotrophin receptor (p75NTR) and Nanog are described cancer stem cells markers in HNSCC. In epithelial cells, p75NTR characterized an immature keratinocyte subset, which was slow-cycling in vivo and presented a strong regenerative potential in vitro, demonstrating the main requirements for stem cells, and introducing the importance of this receptor for the maintenance of a stem cell pool through the induction of a quiescent state. In human adult oral mucosa lamina propria, p75NTR-positive stem cells are self-renewing cells that co-express Oct4 and partially express Sox2 and Nanog transcription factors [42]. In this regard, we have done immunohistochemical stainings in agarose and paraffin embedded SCC-25 and Detroit 562 cells using antibodies against CD-44, Nanog, and p75NTR. Using a combined immunohistochemical reaction for CD-44 (green) and Nanog (red), SCC-25 cells were frequently Nanog positive, but only scattered CD-44 positive (Figure 1A–C). Nearly all Detroit 562 cells were both Nanog and CD-44 positive (Figure 1E–G). Practically, all Detroit 562 cells were p75NTR positive (Figure 1H), while SCC-25 cells were only relatively scattered p75NTR positive (Figure 1D).

### 2.2. MB Effects on Cell Viability with and without Photoactivation

**Figure 2:**
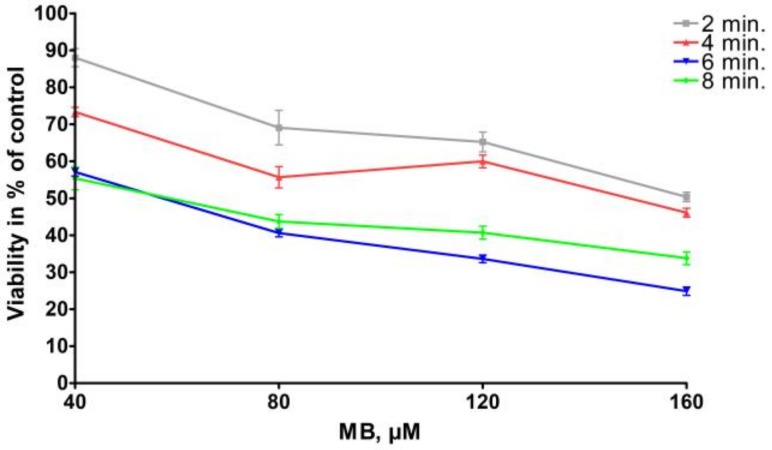
Time and concentration dependent toxic effects of MB on Detroit 562 cells, in percentage of viable cells. MB exposure at concentration of 160 µM for 6–8 min caused up to 75% loss of cells compared to control (sham, scattered light in neighboring wells).

A low level 600 nm diode laser with a constant area dose of 95 J/cm^2^ with up to 15 min exposure caused a nonsignificant loss of cells in HNSCC tumor cell culture, which is comparable with the viability of routine cell culture (Figure 2) [43].

Detroit 562 cells were exposed to increasing concentrations of MB (Table 1) for 2–8 min, without laser exposure. The time conditions were chosen in order to be also reasonable in a clinical oral tumor surface treating, which also meant that we were able to increase the concentration of MB, but we wanted to limit the exposure time. The µM concentrations of MB have been chosen in pre-experiments completed on more cell lines and also on commercial normal fibroblasts. Increasing concentrations of MB caused decreasing cell viability by 2–8 min exposure. The relationship between MB concentration and percentage of control cell viability was linear by exposure times of 2, 6, and 8 min (*r*^2^ = 0.95–0.96), and was not linear by exposure time of 4 min (Figure 2; *r*^2^ = 0.78).

Exposure to a diode laser at 660 nm at a fixed area dose of 95 J/cm^2^ for 2–8 min resulted in a phototoxic effect on top of MB-toxicity (*p* < 0.001) (Table 2). Estimated marginal means of photoxicity when adjusted for the effects of MB-concentration (ranging from 40 µmol/L to 160 µmol/L) and MB-exposure time (ranging from 2 to 8 min) were up to 52% (95%, CI 50% to 55%), MB at 120 µM concentration for 4 min and laser exposure 8 min. For loss of at least 95% of the viable cells 120–160 µM MB was required for at least 4 min, followed by 6–8 min of 660 nm laser exposure. The highest light-depending effect was measured after 120–160 µmol/L 4 min MB treatment followed by 8 min of laser exposure.

**Figure 3:**
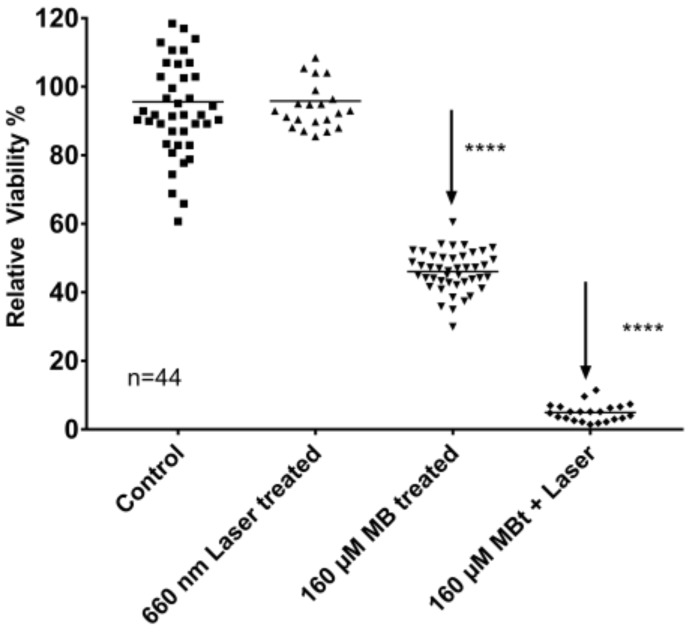
Toxic effects of 160 µM 4 min MB and 8 min laser exposure on Detroit 562 cells. Laser exposure alone ensured 95.8% ± 2.2% cell viability, which was not significantly different from the control (*p* = 0.76 with Mann Whitney test); 160 µM 4 min MB ensured 46.1% ± 0.9% cell viability, which was significantly different from the control (****: *p* < 10^−4^ with Mann Whitney test), the combined MB + 660 nm laser exposure allowed 4.9% ± 0.5% cell viability, which was significantly different from the MB exposure and from the 660 nm laser exposure alone (****: *p* < 10^−4^ with Mann Whitney test).

The phototoxic effect was not monotonically decreasing with increasing laser exposure times. The laser exposure further increased the MB toxicity, 8 min laser exposure allowed at least 95% tumor cell loss after at least 80 µM MB for 4–6 min. The best specific impact for the combination of MB and laser exposure was found in 4 min MB exposure with the concentration of 160 µM and 8 min laser exposure, which allowed up to 95% reduction of the cell viability in comparison to the control (sham, scattered light in neighboring wells). Interestingly, MB alone at concentrations of 120–160 µmol/liter also achieved a 67% reduction of cell viability, when they were treated for 6–8 min. Therefore, the best specific effects were achieved with high concentration of MB, and as short treatment time as possible, and with the maximal laser exposure time. Taking the cell viability results together: since MB alone has an intensive, up to 75% toxicity without light exposure, the MB treatment time was considered to be kept as short as possible by high MB concentrations, and the maximal light-dependent cell toxicity was chosen, which was by 8 min laser exposure. The final preferred conditions: 4 min MB exposure with the concentration of 160 µM and 8 min laser exposure, allowed up to 95% reduction of the cell viability in comparison to the control, and 41% of this cell loss was due to light specific effects and not to MB only (Figure 3).

### 2.3. PDT Effects on Clonogenic Survival

**Figure 4:**
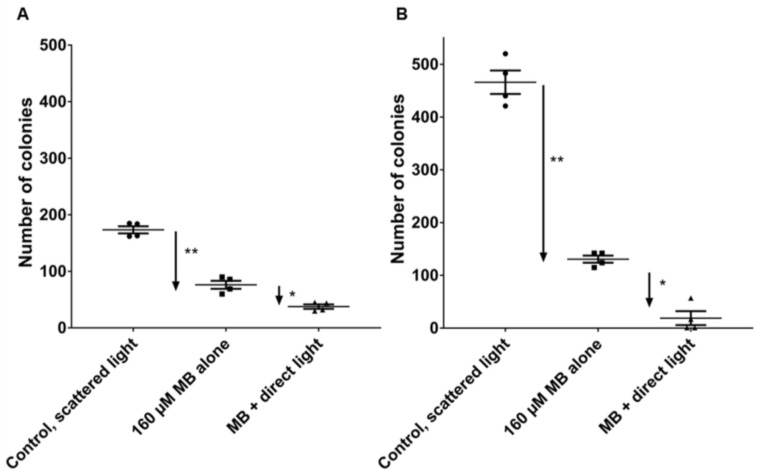
Clonogenic survival of SCC-25 (**A**) and Detroit 562 (**B**) cells after laser and MB treatment. After completion of treatments, 2000 cells/treatment were seeded in 75cm^2^ culture dishes in 15 mL serum-supplemented culture medium for 2 weeks followed by counting the Gentian Violet-stained visible colonies (approximately 50 cells in each colony). In both SCC-25 (**A**) and Detroit 562 cells (**B**) MB significantly reduced (**: *p* < 0.01 by Mann-Whitney test) the number of growing colonies compared to the controls, and MB + laser further significantly (*: *p* < 0.05 by Mann-Whitney test) reduced the number of growing colonies compared to the only MB treated cells, *n* = 4; four biological repeats.

The main interest of the current study is not the analysis of cell viability, which might be a transient reduction of MTT-assay measured mitochondrial and cytoplasmic enzymes [44] but to estimate the tumor reproduction capacity of HNSCC tumor cells after the above-detailed optimized MB and laser treatment conditions. In this regard, the capacity of both SCC-25 and Detroit 562 cells was investigated for producing the numbers of progeny in clonogenic assays. After completion of treatments, 2000 cells/treatment were seeded in 75cm^2^ culture dishes in 15 mL serum-supplemented culture medium for 2 weeks followed by counting the Gentian Violet-stained visible colonies (approximately 50 cells in each colony) (Figure 4). In the culture of SCC-25 cells 173 ± 13 colonies have grown from the plated 2000 cells in conditions of scattered light (control), 160 µM MB alone did not induce significant change in the number of colonies, whereas 160 µM MB (4 min) + 8 min laser treatment high significantly reduced the number of surviving colonies to 38 ± 8 (*p* < 0.01, by Mann-Whitney test, *n* = 4; four biological repeats) (Figure 4A). In the culture of Detroit 562 cells, 466 ± 44 colonies have grown from the plated 2000 cells in conditions of scattered light (control), 160 µM MB alone did not induce significant change in the number of colonies, whereas 160 µM MB (4 min) + 8 min laser treatment high significantly reduced the number of the surviving colonies to 19 ± 26 (*p* < 0.01, by Mann-Whitney test, *n* = 4; four biological repeats) (Figure 4B).

## 3. Discussion

Especially in dermatology PDT is used routinely for patients with malignant skin cancer. PDT with ALA (aminolevulinic acid) is an effective treatment for actinic ceratosis and part of standard treatment. Furthermore, PDT is recommended for squamous cell carcinoma (SCC) in situ, particularly in multiple lesions, where multiple surgeries would be necessary [4,45,46]. Hodgkinson and coworkers [47] reported PDT to be a treatment modality for colorectal cancer using an aid of drug carriers and immune conjugates for enhanced photodynamic therapy efficacy. These modifications could prove effective in targeting cancer stem cells that are thought to be resistant to photodynamic therapy [47]. Another study group described that encapsulation of the photosensitizer MB in nanoparticles leads to an increased production of reactive oxygen species under both normoxic and hypoxic conditions. The authors concluded that nanoparticle encapsulated MB has the capacity to eliminate cancer stem cells under hypoxic conditions, an important goal of current cancer therapy [48]. Also in HNSCC studies could demonstrate PDT to have an effect on HNSCC and has been thoroughly reported in early stage oral cavity tumors. Recently, a Dutch study group matched two groups of patients with early stage oral cavity tumor treated with mTHPC (meta-tetrahydroxyphenylchlorin) mediated PDT (*n* = 55) and treated with surgery (*n* = 43) together, the tumor was thinner than 5 mm. This was the first comparison of PDT to the surgical treatment and PDT was suggested as an alternative to surgery in this tumor state [49]. There are several comparable studies reporting about favorable results for PDT in HNSCC and several photosensitizer such as 3-(1′-hexyloxyethyl) pyropheophorbide [50,51], porfimer sodium [52], mTHPC [53], or 5-aminolevulinic acid [54] have been used to enhance tumor cell death in PDT [55]. Unfortunately, there is less data about PDT with methylene blue in HNSCC. In this study, we found that PDT with methylene blue (MB) and low level laser (LLL) at 660 nm has an effective impact on HNSCC cell lines. Interestingly, methylene blue alone without the use of a laser source could bring a toxic effect on the HNSCC cell lines. In other tumor entities, studies reported the effectiveness of MB-PDT as cancer treatment modality. For melanoma MB-PDT was suggested to be a cheap and efficient method to decrease the volume of malignant melanoma not eligible for surgery. In mice, MB-PDT showed a decrease of 99% in tumor volume and 75% in tumor weight compared with untreated mice (*p* < 0.05) [56]. Previous studies are reporting MB-PDT to induce apoptosis in human lung adenocarcinoma cells. The study group found MB to sensitize A549 cells, adenocarcinomic human alveolar basal epithelial cells to PDT-induced apoptosis. Inter alia the viability assay was used, in contrast to our result MB alone had little effect on cell viability, MB treatment followed by PDT significantly decreased cell viability [57]. According to their results, it was reported that MB was more toxic to leukemia cells then to normal peripheral blood mononuclear cells. This indicates MB to be more toxic in cancer cells than in normal cells [58]. The cytotoxic effect of MB and its derivatives was also proven on murine mammary tumor cell lines, showing a dark toxicity of 7.9% for MB with a concentration of 18.7 µM/L [59]. A cytotoxic effect of MB could also be found on human brain tumor cells suggesting that the treatment of MB may be useful in the therapeutic applications of human brain tumors [60]. In conclusion, our study suggests a significant potential of combined MB-660 nm laser therapy in HNSCC, but MB alone has a remarkable toxicity in HNSCC tumor cell lines. First, the maximal photodynamic effect was assessed. Therefore the best concentration of MB, exposure time of MB, and time of laser exposure were researched. It was considered that the minimal MB exposure time with minimal light exposure with minimal toxicity for tumor cells trough MB alone and the maximal toxicity for tumor cells in combination of MB and light exposure had to be detected to find a reasonable adaptability.

### Toxicity of MB

The American National Institutes of Health and Public Health Service published in 2008 toxicology and carcinogenesis studies of methylene blue trihydrate. Groups of 50 rats received 5, 25, or 50 milligrams of methylene blue trihydrate per kilogram body weight five days per week for two years. Another group of 50 mice received 2.5, 12.5, or 25 milligrams of methylene blue per kilogram of body weight for the same duration. Animals receiving methylcellulose alone served as controls. Exposure to methylene blue caused pancreatic islet tumors in male rats and small intestine tumors in male mice. Malignant lymphomas in male and female mice were possibly associated with methylene blue trihydrate exposure. Methylene blue trihydrate caused blood abnormalities and anemia in male and female rats and mice [61]. Chang and coworker [62] investigated the cytotoxicity of various dyes in corneal endothelial cells in a rabbit model. Structural changes in corneal endothelial cells after dye exposure were evaluated by light microscopy and transmission electron microscopy. MB 0.20% did not induce significant damage to corneal endothelial cells. Significant cytotoxicity was observed with higher dye concentrations, postoperative corneal edema due to endothelial toxicity was caused [62]. Kirszberg and coworker [58] measured the cytotoxicity of MB by MTT assay in erythroleukemic and melanoma lineages, comparing it with normal cells, lymphocytes, and melanocytes. The authors observed that MB was more toxic on erythroleukemic cells than to normal peripheral blood mononuclear cells. The group found that MB was able to inhibit in vitro growth of erythroleukemic cells. The study group concluded MB to be more cytotoxic for tumoral cells and suggests MB to be used as a chemotherapeutic agent [58].

MB exposure at concentration of 160 µM for 6–8 min caused up to 75% loss of cells compared to control (sham, scattered light in neighboring wells).

Exposure to a diode laser at 660 nm at a fixed area dose of 95 J/cm^2^ for 2–8 min resulted in a phototoxic effect on top of MB-toxicity (*p* < 0.001). Therefore, the best specific effects were achieved with high concentration of MB, and as short treatment time as possible, and with the maximal laser exposure time. Taken the cell viability results together: since MB alone has an intensive, up to 75% toxicity without light exposure, the MB treatment time was considered to keep as short as possible by high MB concentrations, and the maximal light-dependent cell toxicity was chosen, which was by 8 min laser exposure. The final preferred conditions: 4 min MB exposure with the concentration of 160 µM and 8 min laser exposure, allowed up to 95% reduction of the cell viability in comparison to the control, and 41% of this cell loss was due to light specific effects and not to MB only. These optimized conditions were used in clonogenic survival assays resulting in significant reduction of growing clones in both SCC-25 and Detroit 562 cells if MB treatment was combined with 8 min 660 nm diode laser exposure. MB treatment alone has shown a visible reduction of the number of colonies, but it was not significant either in cases of SCC-25 or Detroit 562 cells. As visible on Figure 4A,B) Detroit 562 cells have a two-time higher basic cell clone producing capacity than SCC-25 cells, but the MB + laser treatment reduces this capacity to 19 ± 26 colonies, which is lower than that of 38 ± 8 colonies of SCC-25 cells. Immunohistochemical stainings of agarose and paraffin embedded cell pellets of Detroit 562 and SCC-25 cells revealed a high representation of cancer stem cells in the Detroit 562 culture, which is highly consistent with the result that Detroit 562 cell culture has a high clone producing capacity in normal culture conditions. It is particularly interesting, that a short time high concentrated MB treatment followed by 8 min of light exposition, which are, at least from the point of view of time, clinically also endurable, delivered a highly efficient killing of tumorigenic cancer stem cells.

## 4. Materials and Methods

### 4.1. Cell Lines

Detroit 562 cells were purchased from Cell Lines Service (CLS Cell Lines Service, Heidelberg, Germany) and were routinely cultured in DMEM/F12 (PAA) supplemented with 10% FBS (PAA), 2 mM l-glutamine, 100 units/mL penicillin, and 100 μg/mL streptomycin at 37 °C and 5% CO_2_ [39]. SCC-25 cells were acquired from the German Collection of Microorganisms and Cell Cultures (DSMZ, Braunschweig, Germany, DSMZ no.: ACC 617), and were cultured in DMEM/F12 medium, supplemented with 10% FBS, 2 mM l-glutamine, 100 units/mL penicillin and 100 μg/mL streptomycin [34,63,64,65,66].

### 4.2. Evaluation of Cancer Stem Cells—Related Markers in Cultured Cell Lines 

Routinely cultured cell lines (2–4 × 10^6^) were collected by centrifugation and embedded as cell pellet in agarose as published before [67], modified as follows: Cells were harvested by centrifugation at 290 *g* for 10 min at 4 °C, and the resulting pellet was fixed in 10 mL neutral buffered 4% formaldehyde solution (SAV liquid production, Flintsbach am Inn, Germany). After fixation the cells were centrifuged by 400 *g* for 10 min at room temperature. The cell pellet was resuspended in 300 µL PBS, transferred to Eppendorf tube (1.5 mL), and kept on ice. Low melting point agarose (with gelling temperature point 34–37 °C) was prepared in PBS as 3% solution in labor glassware by microwave warming and it was equilibrated in a thermoblock to 65 °C for at least 30 min. The 300 µL PBS—cell suspension was also equilibrated to 65 °C for not more than 10 min. 600 µL melted equilibrated agarose was pipetted to the cell suspension, followed by spinning at 2000× *g* for 5 min at room temperature. After that, the tube was placed on ice, the cell pellet was trimmed and it was placed in embedding cassette. The cell pellet in the cassette was stored in PBS containing 0.05–0.1% sodium azide until embedded in paraffin, which was done using a Histos 5 (Histocom, Wiener Neudorf, Austria) paraffin embedding system, following the instructions of the manufacturer. After embedding, cell pellets were serially sectioned at 5 µm thickness using a HM 355S microtome (Microm, Walldorf, Germany), affixed onto Superfrost^TM^ Plus slides (Menzel, Braunschweig, Germany), and used for immunohistochemistry. Immunohistochemistry was performed utilizing a Ventana Roche^®^ Discovery Immunostainer (Roche, Mannheim, Germany), applying a manufacturer supplied FISH procedure. Antigen retrieval was performed by epitope unmasking via a heat induction methodology performed while the sections were immersed in EDTA buffer (Cell Conditioning Solution CC1, Ventana 950–124; (Roche, Mannheim, Germany). Cell pellet affixed slides were incubated with appropriate primary antibodies (CD44, mouse monoclonal (Antibodies Online, Aachen, Germany, Cat. Nr. ABIN1020059), and Nanog rabbit monoclonal (Cell Signaling Technology; Leiden, The Netherlands, Cat. Nr. 4903)) at 37 °C for 1 h. The primary antibodies were detected with anti-mouse or anti-rabbit Alexa 488 or Alexa 594 conjugated secondary antibodies (Invitrogen, Eugene, OR, USA) incubating for 30 min in the Ventana Discovery immunostainer. In addition mouse monoclonal p75NTR antibody (Sigma, Vienna, Austria, Cat. Nr. N5408) was also used as a single staining detected by Alexa 488 conjugated secondary anti–mouse antibody. The Alexa fluorochrome conjugated secondary antibody signals were detected after 5 min 4′,6-diamidino-2-phenylindole (DAPI) (Invitrogen Eugene, OR, USA) counterstaining on Vectashield mounted slides using fluorescent microscopy. The entire immunohistochemical staining reaction was benchmarked against appositive controls that were supplemented to each experiment. Auxiliary negative controls were acquired by alternating the primary antibodies with reaction buffer or substituting them with isotype matching immunoglobulins. These auxiliary negative controls never yielded any immunostaining.

The antibodies reacted samples were scanned and photographed with Tissue Faxs (Tissue Gnostics Medical & Biotech Solutions, Vienna, Austria). A 2.5× objective was used for the preview and a 20× objective was utilized for the acquisition. As master channel for the focus DAPI was used. The percent of single and double positive cells was identified after scanning the sections in the TissueFaxs system and evaluating with Tissuequest (TissueGnostics, version, Vienna, Austria) software.

### 4.3. MB-Exposure

MB was used as photosensitizer in a 1% solution approved for use in human BLUEbact^®^: 1 mL contained 10 mg of methylthionium chloride and 50 mg of glucose in 1 mL water). MB was chosen as photosensitizer because of the absorption maximum at 660 nm, which was ideal for the used light source (wavelength of 660 nm). This material has been provided by “Heltschl Medizin Technik”, Schlüsslberg, Austria.

The cells were exposed to increasing MB concentrations, several MB exposure times and laser exposure times. On the first day 10^5^ cells/well (10^5^ cells/2 mL medium in DMEM-F12, supplemented with 10% serum) have been plated. On the second day cell media were removed and replaced with medium containing MB in chosen concentrations. After the chosen MB-exposure times the cells were washed twice with 10% serum containing medium. The medium was then replaced trough transparent Leibovitz-15 medium (Lonza, Vienna, Austria, 1 mL/well) and laser-radiation was performed for desired durations. Afterwards, the medium has been replaced with serum containing medium (Full DMEM-F12) again.

### 4.4. Laser Source and Treatment

**Figure 5:**
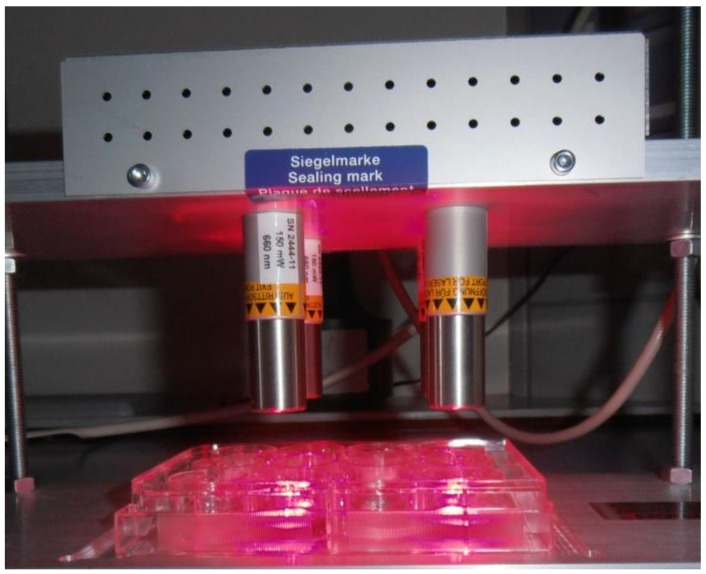
Photodynamic therapy (PDT) exposure after MB treatment. GaAlAs diode area laser sources were used parallel above four wells of a 12-well plate (FL 3500, 660 nm, 350 mW, Heltschl GmbH), 2 cm above the cell culture and the culture dishes were uncovered under the laser source. Control cells in the neighboring wells received scattered light.

GaAlAs diode area laser sources were used parallel above four wells of a 12-well plate (FL 3500, 660 nm, 350 mW, Heltschl GmbH, Schluesslberg, Austria), 2 cm above the cell culture and the culture dishes were uncovered under the laser source (Figure 5). The area laser source has been described by Schartinger and coworker previously [43].

For laser irradiation the Detroit 562 cells were plated in a 12 well cell culture plate at 10^5^ cells/well. For all experiments, a constant area dose of 95 J/cm^2^ was applied. Three factors were systematically varied. MB exposure time of cultured cells was tested for 0 (cells were washed only)/2/4/8/10 min. MB concentration was tested for 0 (glucose 5%)/40/80/120/160 µM/L. Light exposure was tested for 0/2/4/8/10 min. The response-variable was the viability of cells in percent of control.

Twenty-four hours after treatment cells were released from the treatment plates (four wells control, four wells laser and MB exposed, and four wells only MB exposed), and all cells from one well of the 12 well plate were resuspended in 1.2 mL medium and plated in 11 wells of 96 well plate, 100 µL cell suspension/well. The 96 well plate was incubated for 72 h followed by an MMT assay [68].

For further experiments, cell-cultures were exposed to MB with a concentration of 160 µM for 4 min and then to laser light for 8 min. With these settings, the effects on viability (MTT assay) and the clonogenicity were assessed as described.

### 4.5. MTT Assay

Twenty-four hours after MB and laser treatment all cells including controls and only MB-treated cells were removed from the 12-well plates using Trypsin-EDTA (PAA) collecting all wells in separate centrifugation tubes. Cell suspensions were centrifuged by 290 *g* at 4 °C for 5 min, the supernatant was removed and cells in one well of 12-well plate were resuspended in 1.2 mL complete medium. The cell suspension of one well of 12-well plate was distributed in 11 wells of 96 well plate. The plate was incubated for 72 h, followed by cell viability evaluation using MTT-assay as previously described [43].

Briefly: cells were incubated for 4 h at 37 °C and then the formazan reaction product was dissolved using 10% sodium dodecylsulfate in 10 mM HCl at 37 °C for 12 h. Absorbance was read at 550 nm using a microtiter plate reader (Athos 2010, Salzburg, Austria).

### 4.6. Clongenic Assay

Twenty-four hours after MB and laser treatment all cells including controls and only MB-treated cells were removed from the 12-well plates using Trypsin-EDTA (PAA) and collected to 15 mL centrifuge tubes, separate treatments to separate tubes. Cell suspensions were counted using a hämocytometer (Unilab, Innsbruck, Austria) and 2000 cells/treatment were seeded in 75 cm^2^ culture dishes in 15 mL DMEM/F12 supplemented with 10% fetal bovine serum, 2 mM l-glutamin, 100 units/mL penicillin and 100 μg/mL streptomycin (GE Healthcare Cell Culture, Pasching, Austria) for 2 weeks for clonogenic assays. After 14 days of growth the visible colonies (approximately 50 cells in each colony) were visualized by 0.5% Gentian Violet in methanol, which was kept in the culture dishes for 30 s, and differentiated with a gentle stream of lukewarm water.

Stained flasks were scanned in 1200 dpi resolution using a commercial flat-bed scanner. Based upon the resulting micrographs, colonies were counted and occupied areas measured semi-automatically using a macro written in imageJ/FIJI (LOCI in Madison, WI, USA, home of ImageJ2) [69] macro language. Background subtraction was performed on single images using rolling-ball-algorithm. Micrographs were subjected to color deconvolution [70] and filtered using a Fourier band-pass filter. Colonies were segmented using auto-thresholding algorithms. Segmented colonies were counted and occupied areas measured. Resulting data were subjected to statistical analysis, counted were number of colonies by culture dishes.

### 4.7. Statistics

Viability was calculated as percent viable cells or number of colonies under each experimental condition compared with the control conditions by mean comparison using either Student’s *t*-test or Mann-Whitney test depending on the normal distribution of the data.

## 5. Conclusions

MB was used in relatively high concentrations for short time treatments followed by 8 min of 660 nm laser exposure in this study, as an experimental model for PDT for easily accessible oral SCC. MB alone caused a significant toxicity, but in the preferred conditions of 4 min 160 µM MB and 8 min laser exposure 41% of cell viability reduction was light specific. This combination was also particularly effective against cancer stem cells, which were enriched in the multiresistant Detroit 562 cell culture. MB, as we also observed, is relative unselective, for better targeting several research groups try to pack it into nanoparticles as published by Usacheva and coauthors [48]. MB as a PDT agent for the treatment of respiratory tract cancer in animal models was described to be efficient [71]. Further studies are needed to assess the safety and efficacy of MB-associated PDT for the treatment of cancer in humans.

## Figures and Tables

**Table 1:** Time and concentration (µM) dependent toxic effects of methylene blue (MB) on Detroit 562 cells, in percentage of viable cells rounded to integer values.

	MB-Exposure (min)	40	80	120	160
% Viability	2	88	69	65	50
	4	73	56	60	46
	6	57	41	34	25
	8	55	44	41	34

**Table 2:** Phototoxic effect on top of MB-toxicity 660 nm laser exposure after MB pretreatment at different concentrations and times on Detroit 562 cells. MB alone achieved up to 75% cell loss (written in blue). The treatment aim was to decrease the cell viability up to 95% cell loss, which was only achieved in combination of MB and 660 nm laser exposure. The combinations achieved this aim are written in green. The lower part of the table shows the differential effects of 660 nm laser exposure on top of the MB effect. The highest laser-specific effect, which was accompanied with at least 95% cell loss, is written in red.

	660 nm Laser Exposure (min)	MB-Exposure (min)	MB Concentration, µM			
			**40**	**80**	**120**	**160**
% of viable cells	0	2	88	69	65	50
	0	4	73	56	60	46
	0	6	57	41	34	25
	0	8	55	44	41	34
	2	2	66	54	41	31
	2	4	51	39	34	29
	2	6	40	14	10	22
	2	8	28	23	18	12
	4	2	52	39	31	27
	4	4	64	44	35	24
	4	6	30	14	7	3
	4	8	21	14	8	8
	6	2	49	38	30	30
	6	4	69	55	33	25
	6	6	31	6	8	4
	6	8	19	14	7	6
	8	2	46	38	36	27
	8	4	39	8	8	5
	8	6	26	3	2	2
	8	8	19	11	5	3
Viablility % difference to *t* = 0 min Laser exposure	2	2	22	16	24	19
	2	4	22	16	26	17
	2	6	17	27	24	3
	2	8	28	20	23	22
	4	2	36	30	34	23
	4	4	9	12	25	22
	4	6	27	27	27	22
	4	8	35	29	32	26
	6	2	39	31	35	20
	6	4	5	0	27	21
	6	6	26	35	26	21
	6	8	36	30	34	28
	8	2	42	31	30	24
	8	4	34	47	52	41
	8	6	31	38	32	23
	8	8	36	32	36	30

## Abbreviations

5-ALA	5-aminolevulinic acid hydrochloride
APDT	antimicrobial photodynamic therapy
DAPI	diamidino-2-phenylindole
DMEM/F-12	Dulbecco’s Modified Eagle Medium/Nutrient Mixture F-12
EDTA	ethylenediaminetetraacetic acid
FISH	fluorescence in situ hybridization
FBS	fetal bovine serum
HNSCC	head and neck squamous cell carcinoma
HPPH	2-(1-Hexyloxyethyl)-2-devinyl pyropheophorbide
HPV	Human papilloma virus
LLL	low level laser
MB	methylene blue
mTHPC	meta-tetrahydroxyphenylchlorin
MTT	3-(4,5-dimethylthiazol-2-yl)-2,5-diphenyltetrazolium bromide
PBS	Phosphate Buffered Saline
PDT	Photodynamic therapy
PPIX	protoporphyrin IX
SCC	squamous cell carcinoma
SCID	severe combined immunodeficiency
SRB	sulforhodamine B assay
